# Long-term overall survival of testicular cancer: findings from a rural population-based cancer registry, Ratnagiri district, Maharashtra state, India

**DOI:** 10.3332/ecancer.2026.2080

**Published:** 2026-02-24

**Authors:** Monika Sarade, Dipak Das, Sharyu Mhamane, Sandip Bhojane, Suvarna Patil, Shripad Banavali, Gagan Prakash, Atul Budukh

**Affiliations:** 1 Centre for Cancer Epidemiology (CCE), Tata Memorial Centre (TMC), ACTREC, Navi Mumbai 410210, Maharashtra, India; 2 Bhaktashreshtha Kamalakarpant Laxman Walawalkar Hospital, Dervan, Ratnagiri 415606, Maharashtra, India; 3 Tata Memorial Hospital (TMH), Parel, Mumbai 400012, Maharashtra, India; 4 Homi Bhabha National Institute (HBNI), BARC Training School Complex, Anushaktinagar, Mumbai 400094, Maharashtra, India; a https://orcid.org/0009-0003-7768-0665; b https://orcid.org/0009-0008-4113-8870; c https://orcid.org/0000-0002-7406-8134; d https://orcid.org/0000-0003-1722-4337; e https://orcid.org/0000-0001-6723-802X

**Keywords:** testicular cancer, neoplasms, cancer survival, germ cell tumour, cancer registry, rural health, India

## Abstract

**Introduction:**

Tata Memorial Centre (TMC) in collaboration with Bhaktashreshtha Kamalakarpant Laxman Walawalkar Hospital (BKLWH), Dervan, a rural cancer hospital in Ratnagiri, started a population-based cancer registry (PBCR) in Ratnagiri district, Maharashtra, India in February 2009. Testicular cancer survival is a less-explored area of oncology research. This paper aims to present the 10-year survival of testicular cancer patients in Ratnagiri district for the years 2009–2018.

**Methods:**

The registry follows an active method of case registration. Major sources of the cancer registry are TMC and BKLWH. Data quality control was conducted by TMC, Mumbai. Follow-up was performed by house and village visits/phone calls by registry staff. For survival analysis, the date of diagnosis was the starting date, and the last follow-up date was 31 December 2023. The 5-year and 10-year overall survival was calculated using the Kaplan–Meier method and relative survival (RS) by the Pohar Perme method.

**Results:**

A total of 43 testicular cancer cases were registered in Ratnagiri PBCR, with incidence and mortality rates of 0.6 and 0.2 per 100,000 population. The 10-year RS of testicular cancer was 63.3% (95% confidence interval: 45.4–76.7). Survival was 100% for localised cases. Other prognostic factors were age at diagnosis (<40 years), histology (seminoma), ‘surgery’ as a treatment modality and ‘completed’ treatment status.

**Conclusion:**

Good health infrastructure and linkage of rural hospitals with the tertiary cancer centre play a pivotal role in improved testicular cancer outcomes. Continued efforts in research and education for health professionals serving as the first point of contact for the population will be essential to enhance testicular management in India.

## Introduction

Testicular cancer malignancy remains relatively rare. The Globocan estimates the incidence rate of testicular cancer in India to be 0.6 per 100,000 population, while the age-adjusted mortality rate is 0.14 per 100,000 population [1]. Testicular cancer is the most common malignancy among young adult males, particularly affecting individuals aged 15–40 years [2, 3]. Despite its relatively low incidence compared to other cancers, testicular cancer has one of the highest survival rates even in advanced disease, largely due to its response to platinum-based chemotherapy regimen, evolution in complex retroperitoneal surgeries and radiotherapy techniques [4, 5]. This has led to an increase in 5-year survival rates to more than 90% from the previously documented 63% [1, 6]. Survival outcomes for testicular cancer in India are not extensively documented. However, data from high-income countries indicate favorable survival rates. For instance, in Canada, the 5-year net survival for testicular cancer is 97% [7]. Similarly, in the United States, the 5-year relative survival (RS) rate for localised testicular cancer is 99% [8]. According to Cancer Research UK, around 95 out of 100 men (around 95%) will survive their cancer for 5 years or more after diagnosis [9].

Survival outcomes vary significantly across various populations and different geographic regions due to disparities in healthcare access, treatment availability and socioeconomic factors [10]. The Indian context of testicular cancer is less explored; this calls for oncology research to contribute to the literature on testicular cancer, providing insights into risk factors, epidemiology, aetiology and prognosis of the disease. Population-based cancer registry (PBCRs) hold the potential to reflect the true burden of the cancer profile of the given geography. It provides information on the incidence, mortality, prevalence and survival rates, which aid in understanding the effect of cancer care services ongoing in the catchment region [11, 12].

Tata Memorial Centre (TMC), Mumbai, India, a grant-in-aid institute under the Department of Atomic Energy, Government of India, in collaboration with the Bhaktashreshtha Kamalakarpant Laxman Walawalkar Hospital (BKLWH), Dervan, a rural cancer hospital in Ratnagiri district of Maharashtra, India, as well as with the support of district health authorities, started a PBCR in the Ratnagiri district of Maharashtra state, India, in February 2009. The BKLWH, Dervan, is a charitable hospital. It provides cancer diagnostic and treatment services through its excellent infrastructure and prompt referral linkage with the tertiary cancer centre at Tata Memorial Hospital (TMH), Mumbai, which is around 250 km from BKLWH. It is also involved in cancer prevention activities. The senior consultant from TMH, Mumbai, periodically visits BKLWH, Dervan, for capacity building and providing treatment consultations.

**Figure 1.**
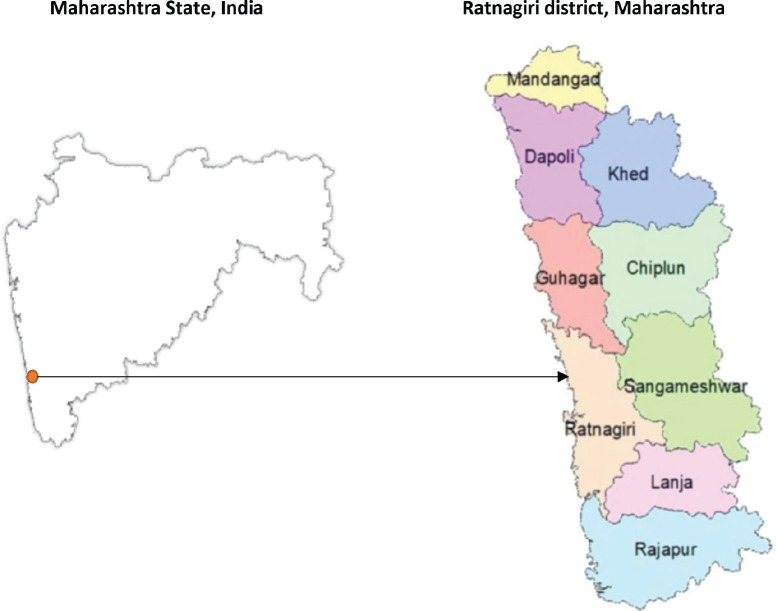
Registry area covered by the population-based cancer registry Ratnagiri district, Maharashtra, India.

The Ratnagiri PBCR covers nine subdivisions of the district – Chiplun, Guhagar, Lanja, Rajapur, Sangameshwar, Dapoli, Khed, Mandangad and Ratnagiri, covering a population of 1,615,069. The registry covers urban areas as well as 1,537 villages in the district. The registry area is presented in Figure 1. More than 80% of the population of the district is rural [13]. There is limited data on survival from low- and middle-income countries (LMICs) as well as from rural areas; therefore, there is a need for literature on cancer statistics from these regions. Through this study, we aim to present the long-term survival of testicular cancer patients registered in Ratnagiri PBCR from the state of Maharashtra, India, for the years 2009–2018 [14]. This paper provides insights into prognostic factors responsible for dictating the testicular cancer outcomes and its management in a rural cancer registry of India.

## Methodology

The PBCR uses an active method of cancer case registration. To gather data on cancer incidence and death cases, registry-trained social workers routinely visit hospitals, pathology labs, medical colleges, cancer control cells and the birth and death registrar's office. The trained registry staff verifies these cases obtained from the hospital/medical facilities through field visits and registers the cancer cases by communicating with patients or their family members and records the available cancer case data with the assistance of an accredited social health activist (ASHA). In addition to the medical and diagnostic facilities, the registry staff periodically communicates with the village sarpanch (village head), auxiliary nurse midwife, ASHA workers and primary health centre staff to obtain information about newly diagnosed cancer cases as well as cancer deaths that occurred in the community. For the cases obtained during field visits, the information is further confirmed at the patient’s treating hospital.

For Ratnagiri PBCR, TMH, Mumbai and BKLWH, Dervan are two major sources of cancer case information, along with hospitals from Ratnagiri, Karad, Miraj and Kolhapur [13, 14]. As it is a major cancer centre in Ratnagiri, more than 85% of cases in Ratnagiri are recorded in this hospital.

The concept of PBCR includes only those cases that are residents of the geographical location from the past 1 year and it gives the true burden of the disease occurring in the geography. After confirming the patient’s residence (resident of the district for at least 1 year) and duplicate checking and applying the quality control measures by senior staff from TMH, Mumbai, the case is registered in the prescribed format. The data are coded as per the International Classification of Diseases for Oncology, third edition guidelines [15]. The data are entered into the CanReg5 software developed by the World Health Organisation-International Agency for Research on Cancer (IARC), Lyon, France [16]. After all quality measures, the cancer incidence and death cases are registered. The staff at the Centre for Cancer Epidemiology, TMC (CCE-TMC) checks all the data and ICD coding, as well as the difficult cases are discussed with the clinician/pathologist. The CCE-TMC team regularly visits the project office at Dervan, Ratnagiri, for quality control.

When the treatment source was missing, information was gathered through interaction with the patient's family, and treatment-related data was gathered. The majority of clinical data are obtained from TMH and BKLWH, while a few private hospitals refuse to share data. In such cases, the registry staff conducts house visits and collects cancer case information from the patients/patient relatives. Cancer diagnosis and treatment facilities in some private hospitals and pathology labs are available in the district. Some patients visit nearby areas, Miraj, Kolhapur, Karad and Mumbai for further investigation and treatment of cancer. Therefore, these sources were also traced to register the cancer cases and treatment-related details from the PBCR area. The senior consultant from TMH, Mumbai, visiting BKLWH, Dervan, ensures optimum care and treatment for the cancer patients attending the hospital from these areas. In recent years, health schemes such as the Mahatma Jyotiba Phule Jan Arogya Yojana and Ayushman Bharat – Pradhan Mantri Jan Arogya Yojana cards provide healthcare cover for cancer-based treatments. These schemes are instrumental in cancer treatment completion and alleviate the financial burden accrued to cancer patients during the treatment [17], therefore, contributing to improved survival of the cancer patients in Ratnagiri district.

As part of the registry methodology, the registry staff updates the current vital status of cancer case information through regular follow-up conducted through community engagement and regular house and village visits/phone calls to cancer patients. The registry was able to update the survival status of cancer patients. The database was updated annually. Survival status was confirmed by a combination of active follow-up approaches, including home visits and telephone calls, as well as passive follow-up via hospital records and linkage to the municipal death registry. No instances were documented as having migrated out of the registry region throughout the follow-up period in our dataset. Consequently, no further modifications or protocols for migration data were necessary for this investigation.

For testicular cancer survival analysis, the date of diagnosis was taken as the starting date, and the last follow-up date is December 31, 2023. The survival time was calculated in years between the starting and the closing date. Survival data for the years 2009–2018 were entered into Microsoft Excel and analysed using STATA version 15.0 (Stata Corp, College Station, TX, USA) [18]. The Kaplan–Meier method was applied to calculate the overall survival rates at 5 and 10 years [19, 20]. RS was calculated employing the Pohar–Perme approach to consider background mortality [21, 22]. We utilised the rural Maharashtra life table as the reference population for the RS analysis. A Cox proportional hazards regression model was utilised to discover independent determinants of survival [23]. Additionally, age-standardised survival was conducted to facilitate significant comparisons of RS among different populations [22]. We used the ‘sts’ command for Kaplan–Meier survival estimates, ‘stcox’ for Cox regression models and ‘strs’ for RS analysis — each of which was developed by Paul Dickman for use in Stata [22].

In this study, censored data were not considered in the analysis. All censored cases were excluded from both the Kaplan–Meier survival estimation and the Cox proportional hazards modelling. This is a regular public health service and routine practice conducted with the help of district health authorities and the state government; therefore, informed consent was not required to collect data.

### Data availability statement

The authors confirm the availability of, and access to, all original data reported in this study.

## Results

For the years 2009–2018, a total of 43 testicular cancer cases were registered in Ratnagiri PBCR. The incidence and mortality rate of testicular cancer for the Ratnagiri cancer registry were 0.6 and 0.2 per 100,000, respectively. The majority of the cases are confirmed with histopathology. Table 1 presents the characteristics of testicular cancers registered at the Ratnagiri PBCR (2009–2018). The majority (88.4%) of cases were from TMH and BKLWH. Around 80% of cases had an age of less than 40 years at the time of cancer diagnosis. Of the total cases, 27.9% were seminomas, 60.5% were non-seminomas and 11.6% were classified as others. 37% of the cases were diagnosed at the loco-regional stage (Stage II/Stage III). Among treatment modalities, ‘Surgery in combination with Chemotherapy’ was advised for 58.1% of the cases. Of the total cases, 88.4% completed treatment. As per the registry principle, ‘Treatment completed’ is the term used for updating the treatment status of the patient who has completed the treatment prescribed by the doctor. While ‘incomplete treatment’ is the term used for patients who did not or could not complete the prescribed treatment, this can be either due to the decision to opt out of the prescribed treatment, or the death of the patient during or before the completion of the prescribed treatment.

The table also describes the type of surgical intervention in the surgery component of the treatment modality. The majority of the surgical interventions were orchiectomy; other interventions include hemicolectomy or Retro-Peritoneal Lymph Node Dissection (RPLND) combined with orchiectomy. Additionally, the table describes stage-wise treatment completion status. Treatment completion was 100% for localised and distant metastatic cases, while only 75% of loco-regional cases had completed the prescribed treatment. The details are presented in Table 1.

The age-standardised RS is presented in Table 2. For all age‑group and age groups of 0–79 years, the age‑standardised 5‑year RS was 71% [95% CI: 54.3–82.3] and 71.8% [95% CI: 55.3–82.3], respectively, while the 10‑year RS was 63.3% [95% CI: 45.4–76.7] and 62.8% [95% CI: 44.2–74.2], respectively. For all age groups, the 1 and 3‑year age-standardised RS was 81.6% [95% CI: 66.3–90.5] and 72.8% [95% CI: 56.4–83.7], respectively.

**Figure 2.**
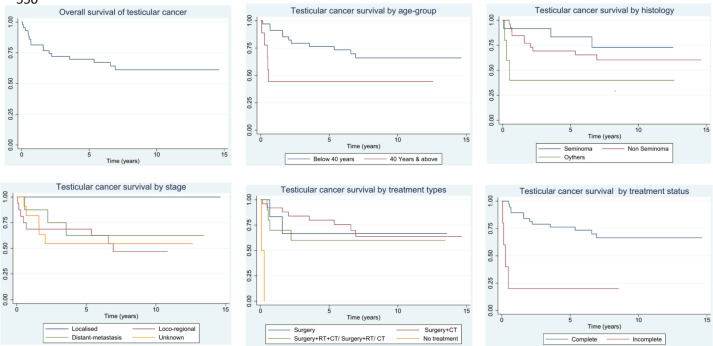
Overall survival, survival by age group, histology, treatment types and treatment status of testicular cancer patients in Ratnagiri district (2009–2018).

The results of the observed and RS of testicular cancer patients by selected background characteristics are presented in Table 3. The RS of testicular cancer was higher among cases diagnosed before the age of 40 (67.4%) (95% CI: 47.3–81.2) as opposed to those diagnosed at age ≥40 years had relatively lower RS (45.8%) (95% CI: 14.1–73.4). Interesting observations for stage-related characteristics were observed, with 100% survival of localised cases, followed by 63% (95% CI: 22.7–87.3) for distant metastasis and 47% (95% CI: 20.0–70.4) for locoregional stage. This is because 100% of cases in localised and distant metastatic cases completed the treatment. However, the treatment completion of locoregional is comparatively low. Based on the histological characteristics, 10-year RS was the highest for Seminomas (77.1%) (95% CI: 33.0–94.1), followed by non-seminoma (61.6%) (95% CI: 39.0–77.8) and others (40.1%) (95% CI: 05.3–76.0). Survival outcomes were the highest (67.5%) (95% CI: 17.4–93.3) with ‘Surgery’ as the treatment modality. Similarly, survival outcomes were superior among cases that completed the prescribed treatment (68.8%) (95% CI: 49.3–82.1). The overall survival of testicular cancer patients, along with survival by age group, histology, treatment type and treatment status, is presented in Figure 2. In the data, it was observed that those cases who completed the advised treatment and regular follow-up performed had better survival across all the characteristics. All localised cases underwent the advised treatment and were on regular follow-up.

The results of the Cox proportional hazard model of testicular cancer patients by selected background characteristics show that the survival outcomes varied between histology, stage at diagnosis and treatment types, but the results were not statistically significant. However, for the age at diagnosis, the survival of testicular cancer was lower among patients with age more than 40 in contrast to those less than the age 40 at the time of diagnosis, and the results were statistically significant (Hazard ratio (HR: 2.9) 95% CI: 1.01–8.54). Similarly, results were statistically significant for treatment status, where those with incomplete treatment had poor survival outcomes (HR: 7.5, 95% 2.36–24.17] than their respective counterpart. Details are presented in Table 4.

## Discussion

To the best of our knowledge, this is the first population-based study in India reporting the long-term (10-year) survival of testicular cancer from a rural population-based registry of Maharashtra, India. In this population-based study from the Ratnagiri Cancer Registry, we examined the ten-year survival outcomes of testicular cancer patients, providing valuable insights into prognostic factors and potential areas for improvement in disease management.

We observed a 10-year RS rate of 63.3% for testicular cancer patients. This was comparatively lower than the testicular cancer survival observed in Western countries. These findings contrast with data from high-income countries, where testicular cancer generally has a more favourable prognosis. For instance, in England, approximately 90% of men survive their cancer for ten years or more after diagnosis (Cancer Research UK, 2023). Similarly, in the United States, the 5-year RS rate for testicular cancer is about 95% (American Cancer Society, 2023). The lower survival rates observed in our study may be attributed to factors such as delayed diagnosis and limited access to specialised care. The Ratnagiri PBCR has reported having excellent diagnostic and treatment facilities at both of its major sources of cancer registration, i.e., TMH, Mumbai and BKLWH, Dervan [14]. In rural settings, such infrastructure and its appropriate linkage with the tertiary cancer centre are essential. Though the survival rates of testicular cancer are inferior compared to the West, our study reported 100% survival for the localised cases. This might be due to a lower number of cases, as well as 100% of cases completed the advised treatment in the category. The survival of localised testicular cancer cases is consistent with the existing literature, suggesting better survival of testicular cancer at early stages in high-income countries such as the US and the UK [9].

In the Indian context, studies have reported varying survival outcomes. Additionally, this study deviates from hospital-based literature, reiterating the substantial decrease in survival with increasing stages of testicular cancer [24–27]. In this study, obtaining data on the stage of cancer is a difficult task for PBCR. The population-based survival study conducted by the IARC WHO reported that 37% of cancer registries have not provided the staging data [28]. The present study shows; more than 50% cases are staged as loco-regional/distant metastasis. However, in this study, the treatment completion status is 100% complete for localised and distant metastatic cases, whereas 10% of the rural population did not complete the prescribed treatment; therefore, the survival is better as compared to the loco regional cases, as only 75% cases had complete treatment status for loco-regional cases, thus affecting the survival.

Patients diagnosed before the age of 40 exhibited a higher RS rate of 67.4%, whereas those diagnosed at 40 years or older had a significantly lower rate of 45.8%. This indicates that the early age of diagnosis played an important role in dictating the survival outcomes of the patient with testicular cancer. Age at diagnosis significantly influences survival outcomes. Our observation that younger patients have better survival aligns with existing literature [29–32]. Testicular cancer is most common in young adults, and early detection and prompt medical attention in this group contribute to higher survival rates [33].

The histological subtype also plays a crucial role in determining prognosis. Histological analysis in our study revealed that seminomas had the highest ten-year RS at 77.1%, followed by non-seminomas at 61.6%, and other histological types at 40.1%. A retrospective study from a tertiary cancer centre in Eastern India reported a 4-year overall survival rate of 87.1% for non-seminomatous germ cell tumours [34]. Another study from Northeast India found that the median recurrence-free survival was 43 months, with better outcomes observed in seminomas compared to non-seminomas [35]. In our study, 11.6% of patients were classified as “others” which is the observed distribution in our setting and remains consistent with the other population-based cancer registries, in different locations of India [36].

There are fewer population-based studies on testicular cancer survival, which limits the comparison of survival rates with other LMICs. In Sudan, a resource-limited setting, a hospital-based study revealed a concerning high mortality rate from testicular cancer. Late presentation and advanced-stage disease at diagnosis were significant contributors to poor outcomes. The study emphasised the necessity for targeted health education campaigns and efficient referral pathways to promote early presentation and access to care [37]. Another study from Brazil reports that five-year survival rates in Brazil varied by histological type, ranging from 48% to 92% [38]. Regarding treatment modalities, surgical intervention was associated with the highest survival rate in this population. This finding underscores the importance of appropriate surgical management in testicular cancer. It is well-established that multimodal treatment approaches, including surgery, chemotherapy and radiotherapy, contribute to high cure rates, with five-year survival rates exceeding 95% in many cases [39, 40]. In our study, surgical intervention (orchiectomy was a majorly performed surgery component in the treatment modality) for testicular cancer was associated with the most favourable survival outcome, with a 10-year RS rate of 67.5%.

Our findings contribute to the growing body of literature on testicular cancer survival, particularly in low- and middle-income settings, where data remains limited. Through this study, findings deduce that age at diagnosis and treatment completion status are important prognostic factors when it comes to long-term survival of testicular cancer. The disparities in survival rates between our study and other Indian studies may be due to differences in healthcare infrastructure, patient awareness and accessibility to specialised care.

Addressing these challenges is essential. Though the incidence rate of testicular cancer is low, there is a threat that poses to the quality of life of men during survivorship [5, 41, 42].

In rural areas, the first point of contact is the primary health centres; therefore, Continuing Medical Education should be conducted to spread awareness about the risk factors, early diagnosis and treatment necessary to tackle testicular cancer. This will be instrumental in early detection and management, consequently leading to enhanced survival outcomes for testicular cancer patients in a region. One notable factor contributing to improved survival in our population is the availability of high-quality cancer treatment at BKLWH in Ratnagiri, which is achieved through the good infrastructure of BKLWH and the technical support of TMH, Mumbai. Additionally, there exists a good referral linkage between BKLWH and TMH, Mumbai. The periodical consultation of medical experts from TMH, Mumbai, to BKLWH has helped in bridging the cancer care gap between a rural and a tertiary cancer centre. This practice has ensured that patients receive standardised and evidence-based management, which may have contributed to better survival outcomes among those who completed their prescribed treatment. This strategy of strengthening such healthcare facilities and ensuring continued access to expert supervision can further enhance survival outcomes. Despite the availability of high-quality treatment, one of the major challenges remains delayed presentation and diagnosis. Many patients may not seek medical attention promptly due to a lack of awareness, stigma or financial constraints. Given that younger age at diagnosis is associated with better prognosis and favourable survival outcomes, increasing awareness and education among primary health care workers in the rural setting will be helpful in prompt referral, thereby leading to early diagnosis and medical attention to testicular cancer symptoms.

PBCRs play an important role in providing cancer control services. About 15% of the Indian population is covered by the cancer registries [43]. There exists a need to improve and expand this coverage by establishing more cancer registries in India. While survival outcomes in our study are lower compared to high-income countries, strengthening cancer registries, improving healthcare infrastructure and implementing targeted public health initiatives can contribute to better prognosis and survival rates in the region. It is reported that an update of follow-up is better in the rural setting [10]. Ratnagiri PBCR has ensured optimum follow-up of cancer patients, which has helped in understanding the survival of cancer patients in Ratnagiri. This was achieved through community interaction and sustained efforts of the registry.

This study has multiple limitations. The sample size is limited due to the rare epidemiology of testicular cancer in the registry region, as well as other Indian registries. As per the Cancer Incidence in Five Continents (CI5) 12th Edition, the range of testicular cancer incidence was from AAR 6.4 per 100,000 in Europe and 0.14 per 100,000 population in Asia. For Indian registries, it ranged from 0.2 to 0.8 per 100,000 population [36]. The limited sample size constrained the statistical power and accuracy of estimations, especially in multivariable models. Second, approximately 25% of the cases presented with an indeterminate stage at diagnosis, hence limiting the analysis of stage-specific survival outcomes. Third, one category in the Cox proportional hazards model lacked occurrences (e.g., localised stage), leading to their omission from the study and constraining the thoroughness of comparisons across all clinical categories. Future research utilising larger datasets and more comprehensive clinical information is advised to corroborate and enhance these findings.

Ratnagiri PBCR is a well-established registry, and BKLWH is a major cancer centre in the region that is linked to TMH, Mumbai. This ensures prompt cancer case registration. The chances of under-reporting and under-diagnosis are less than 10%. It has also yielded favourable survival outcomes for testicular cancer cases. However, this study limits the representation of its outcomes to other rural settings. It can be achieved with adequate linkage between the rural cancer centre and the tertiary cancer centre.

## Conclusion

This study provides valuable insights into the 10-year survival outcomes of testicular cancer patients in the Ratnagiri region, highlighting the impact of age at diagnosis, histological subtype and treatment modality on survival rates. The findings state that treatment played an important role in dictating the survival outcomes of testicular cancer cases. There exists excellent referral linkage between TMH, Mumbai and BKLWH, Dervan in addition to the support received from the consultants of TMH, Mumbai. The rural cancer centres, for effective cancer control and prevention, should be linked to a tertiary cancer centre, bridging the care gap between the two regions.

## List of abbreviations

ASHA: Accredited Social Health Activist; BKLWH: Bhaktashreshtha Kamalakarpant Laxman Walawalkar Hospital; CCE: Centre for Cancer Epidemiology; HR: Hazard Ratio; LMICs: Low- And Middle-Income Countries; PBCR: Population-Based Cancer Registry; RPLND: Retro-Peritoneal Lymph Node Dissection; RS: Relative Survival; TMC: Tata Memorial Centre; TMH: Tata Memorial Hospital.

## Conflicts of interest

The authors declare no conflicts of interest.

## Funding

Nil.

## Author contributions

MS: Data management, data quality control, reviewing and editing

DD: Data management, data preparation, data analysis, data quality control, data visualisation

SM: Literature review, writing the first draft of the manuscript and data visualisation

SB: Data collection, data quality control and assistance in writing the manuscript

SP: Reviewing and editing, assistance in writing the manuscript

SB: Reviewing and editing, assistance in writing the manuscript

GP: Reviewing and editing, assistance in writing the manuscript

AB: Conceptualisation, overall supervision, reviewing and editing the manuscript

## Ethical approval

This is a regular public health programme run with the help of the district health authorities of the state government; therefore, a separate ethical approval was not required for this study.

## Figures and Tables

**Table 1.** Characteristics of testicular cancers registered at the Ratnagiri PBCR (2009–2018).

Background characteristics of cancer cases registered		
Characteristics	*N*	%
**Source**		
TMH+BKLW	38	88.4
Other hospitals	5	11.6
**Age at diagnosis**		
<40 year	34	79.1
≥40 year	9	20.9
**Histology**		
Seminoma	12	27.9
Non-seminoma	26	60.5
Others	5	11.6
**Stage**		
Localised	8	18.6
Loco-regional	16	37.2
Distant-metastasis	8	18.6
Unknown	11	25.6
**Treatment types**		
Surgery	6	14.0
Surgery+CT	25	58.1
Surgery+RT+CT/ Surgery+RT/ CT	10	23.3
No treatment	2	4.7
**Treatment status**		
Complete	38	88.4
Incomplete	5	11.6
**Vital status**		
Alive	27	62.8
Dead	16	37.2
Total	**43**	**100.0**

*Type of surgical intervention for the surgery component of treatment modality*			
Treatment	Type of surgery		
	Orchiectomy *N* (%)	Orchiectomy + others* *N* (%)	Grand total
Surgery	5 (83.3%)	1 (16.4)	6
Surgery+RT	2 (100%)	-	2
Surgery+CT	23 (92%)	2 (8%)	25
Surgery+RT+CT	2 (100%)	-	2
Total	32 (91.4%)	3 (8.6%)	35

Treatment status as per the stage distribution				
Stage distribution	Treatment completion status			
	Completed N (%)	Not Completed N (%)	Treatment not taken/ Unknown / No Information N (%)	Grand Total
Localization	8 (100%)	-	-	8
Loco-regional	12 (75%)	2 (12.5%)	2 (12.5%)	16
Distant metastasis	8 (100%)	-	-	8
Unknown / No Information	10 (90.9%)	1 (9.1%)	-	11
**Total**	38 (88.4%)	3 (7.0%)	2 (4.7%)	43

* *Other include RPLND and Hemicolectomy*

**Table 2:** Age‑standardised overall relative survival for patients diagnosed with testicular cancer.

Age-standardised overall relative survival	1 Year (95%CI)	3 Year (95%CI)	5 Year (95%CI)	10 Year (95%CI)
**Relative survival (all ages)**	81.6 (66.3, 90.5)	72.8 (56.4, 83.7)	71.0 (54.3, 82.3)	63.3 (45.4, 76.7)
**Age-standardised relative survival (all ages)**	81.6 (66.3, 90.5)	72.8 (56.4, 83.7)	71.0 (54.3, 82.3)	63.3 (45.4, 76.7)
**Age-standardised relative survival (0–79)**	82.2 (69.0, 89.9)	73.6 (57.7, 83.6)	71.8 (55.3, 82.3)	62.8 (44.2, 74.2)

**Table 3:** Results of the observed and relative survival of testicular cancer patients by selected background characteristics

Characteristics	Observed Survival (%)				Relative Survival (%)			
	1 Year (95%CI)	3 Year (95%CI)	5 Year (95%CI)	10 Year (95%CI)	1 Year (95%CI)	3 Year (95%CI)	5 Year (95%CI)	10 Year (95%CI)
**Age at diagnosis**								
**< 40 year**	91.2 (75.1, 97.1)	79.4 (61.6, 89.6)	76.5 (58.4, 87.5)	65.9 (46.9, 79.7)	91.4 (74.9, 97.2)	79.9 (61.8, 90.1)	77.3 (58.8, 88.3)	67.4 (47.3, 81.2)
**≥ 40 year**	44.4 (28.2, 87.8)	44.4 (13.6, 71.9)	44.4 (13.6, 71.9)	44.4 (13.6, 71.9)	44.5 (14.0, 72.1)	45.8 (14.03, 73.3)	45.8 (14.1, 74.6)	45.8 (14.1, 73.4)
**Histology**								
**Seminoma**	91.7 (53.9, 98.7)	91.7 (53.9, 98.7)	83.3 (48.2, 95.6)	72.2 (36.8, 90.5)	92.1 (51.7, 98.9)	93.2 (44.9, 99.4)	85.9 (45.2, 97.1)	77.1 (33.0, 94.1)
**Non-seminoma**	84.6 (64.4, 93.9)	69.2 (47.8, 83.3)	69.2 (47.8, 83.3)	60.4 (38.6, 76.5)	84.8 (64.0, 94.1)	69.6 (47.9, 83.7)	69.9 (48.1, 83.9)	61.6 (39.0, 77.8)
**Others**	40.0 (05.2, 75.3)	40.0 (05.2, 75.3)	40.0 (05.2, 75.3)	40.0 (05.2, 75.3)	40.1 (05.4, 75.1)	40.1 (05.3, 75.3)	40.1 (05.4, 75.6)	40.1 (05.3, 76.0)
**Stage**								
**Localised**	100.0	100.0	100.0	100.0	100.0	100.0	100.0	100.0
**Loco-regional**	68.8 (46.3, 89.8)	68.8 (40.5, 85.6)	68.8 (40.5, 85.6)	45.8 (20.8, 69.4)	68.9 (40.6, 85.8)	69.3 (40.7, 86.1)	69.3 (40.7, 86.5)	47.1 (20.0, 70.4)
**Distant-metastasis**	87.5 (38.7, 98.1)	75.0 (31.5, 93.1)	62.5 (22.9, 86.1)	62.5 (22.9, 86.1)	87.7 (38.0, 98.2)	75.5 (31.1, 93.4)	63.2 (22.8, 86.7)	63.2 (22.7, 87.3)
**Unknown**	81.8 (44.7, 95.1)	54.6 (22.9, 77.9)	54.6 (22.9, 77.9)	54.6 (22.9, 77.9)	82.2 (44.1, 95.)	55.6 (23.2, 79.0)	56.0 (23.2, 80.0)	56.0 (23.3, 81.8)
**Treatment Types**								
**Surgery**	83.3 (26.9, 99.4)	66.7 (20.3, 85.7)	66.7 (20.3, 85.7)	66.7 (20.3, 85.7)	83.6 (26.9, 97.6)	67.5 (19.4, 91.0)	67.5 (19.0, 91.5)	67.5 (17.4, 93.3)
**Surgery+CT**	92.0 (51.5, 98.6)	84.0 (47.2, 95.6)	80.0 (61.8, 90.1)	63.7 (22.6, 87.3)	92.3 (70.9, 98.1)	84.8 (62.6, 94.3)	81.3 (58.5, 92.3)	65.4 (39.6, 82.3)
**Surgery+RT+CT/ Surgery+RT/ CT**	70.0 (31.4, 93.1)	60.0 (23.2, 79.4)	60.0 (23.2, 79.4)	60.0 (23.2, 79.4)	70.2 (33.0, 89.3)	60.6 (25.4, 83.2)	60.6 (25.4, 83.7)	60.6 (25.3, 84.9)
**No treatment**	0.0	0.0	0.0	0.0	0.0	0.0	0.0	0.0
**Treatment Status**								
**Complete**	89.5 (74.3, 95.9)	79.0 (62.3, 88.8)	76.3 (59.4, 86.9)	66.4 (48.2, 79.6)	89.7 (74.2, 96.1)	79.7 (62.6, 89.6)	77.6 (60.0, 88.2)	68.8 (49.3, 82.1)
**Incomplete**	20.0 (0.84, 58.2)	20.0 (0.84, 58.2)	20.0 (0.84, 58.2)	20.0 (0.84, 58.2)	20.1 (02.0, 19.2)	20.1 (02.7, 49.4)	20.1 (02.7, 49.6)	20.1 (02.7, 50.1)
**Overall**	**81.4** (66.2, 90.2)	**72.1** (56.1, 83.1)	**69.8** (53.7, 81.2)	**61.1** (44.3, 74.3)	**81.6** (66.3, 90.5)	**72.8** (56.5, 83.8)	**71.0** (54.4, 82.4)	**63.3** (45.4, 76.7)

**Table 4:** Cox proportional hazard model of testicular cancer patients by selected background characteristics

Characteristics	Alive (*n*)	Death (*n*)	Total (*n*)	HR	*p*-value	95% CI	
**Age at diagnosis**							
< 40 year	23	11	34	1.0			
≥ 40 year	4	5	9	2.9	0.048	1.01	8.54
**Histology**							
Seminoma	9	3	12	1.0			
Non-seminoma	16	10	26	1.7	0.442	0.46	6.04
Others	2	3	5	4.7	0.061	0.93	23.48
**Stage**							
Localised*	8	0	8	2.4			
Loco-regional	8	8	16	1.0			
Distant-metastasis	5	3	8	2.3	0.532	0.17	2.47
Unknown	6	5	11	0.9	0.867	0.30	2.79
**Treatment Types**							
Surgery	4	2	6	1.0			
Surgery+CT	17	8	25	0.9	0.884	0.19	4.21
Surgery+RT+CT/ Surgery+RT/ CT	6	4	10	1.2	0.812	0.22	6.72
No treatment*	0	2	2				
**Treatment Status**							
Complete	26	12	38	1.0			
Incomplete	1	4	5	7.5	0.001	2.36	24.17
Total	27	16	43				

* **Omitted from the Cox proportional hazards model due to the absence of events in either the alive or death category.**
